# Enteroviral meningitis reduces CSF concentration of Aβ42, but does not affect markers of parenchymal damage

**DOI:** 10.1007/s10096-019-03569-0

**Published:** 2019-05-15

**Authors:** Kacper Toczylowski, Malgorzata Wojtkowska, Artur Sulik

**Affiliations:** 1 Department of Pediatric Infectious Diseases, Medical University of Bialystok, Waszyngtona 17, 15-274 Bialystok, Poland; 2 Department of Pediatric Laboratory Diagnostics, Medical University of Bialystok, Waszyngtona 17, 15-274 Bialystok, Poland

**Keywords:** Enteroviral meningitis, Children, Meningitis, Biomarkers, Amyloid, Tau proteins

## Abstract

Biomarkers classically studied in Alzheimer’s disease have been analyzed in numerous central nervous system infections in adults, but there are scarce data on these biomarkers in children. Enteroviruses appear to be the most common cause of aseptic meningitis throughout the world. The aim of the study was to investigate neuroinflammatory properties of non-polio enteroviruses by measuring CSF concentrations of biomarkers that are involved in neuropathological pathways of neurodegenerative disorders. We measured Aβ42, t-tau, and S100B concentrations in 42 children with enteroviral meningitis (EM) compared to control group without central nervous system infection. We found enteroviral meningitis (EM) to reduce CSF concentration of Aβ42 (median, 1051.1 pg/mL; interquartile range (IQR), 737.6–1559.5 vs. median, 459.4 pg/mL; IQR, 312.0–662.0, *p* < 0.001). In contrast, CSF concentrations of t-tau and S100B were not affected by EM. There was a correlation between total neutrophil count in CSF and Aβ42 (*R* = − 0.59, *p* < 0.001). Absolute number of mononuclear cells in the CSF correlated with CSF t-tau (*R* = 0.41, *p* < 0.05). Both correlations remained significant after adjustment for age, blood leukocytes, serum CRP, CSF leukocytes, and CSF protein concentration.

## Introduction

Enteroviruses are ubiquitous, and most individuals encounter at least one of these viruses at some time in their lives. The overwhelming majority of viral meningitis cases of any ages are caused by non-polio enteroviruses. Given non-polio enteroviruses are so common and they are known to exhibit strong neurotropism, it is well worth analyzing brain injury following enteroviral central nervous system (CNS) infection.

Biomarkers classically studied in neurodegenerative disorders such as Alzheimer’s disease (AD) are being investigated in CNS infections of bacterial and viral origin [1, 2]. Traditionally AD is characterized by accumulation of amyloid β plaques and neurofibrillary tangles [3]. The two hallmark features still leave a gap in the understanding of AD pathogenesis. Substantial evidence now indicates that inflammation is playing a key role in AD [4]. Over the past decades, a number of infectious agents, including viruses of Herpesviridae family, have been postulated to be involved in AD etiopathogenesis [1, 5]. To date, little is known on enteroviruses influencing inflammatory pathways similar to those seen in AD. Currently, S100B is another emerging biomarker of blood–brain barrier (BBB) permeability and CNS injury. S100B is a calcium-binding protein produced predominantly by astrocytes in the brain. Elevated concentrations of CSF S100B were measured in various CNS pathologies [6, 7]. Interestingly, S100B levels in body fluids are elevated prior to clinical symptoms and imaging findings and may predict disease outcome or treatment efficacy [6, 8].

In the current study, we aimed at investigating neuroinflammatory properties of non-polio enteroviruses by measuring CSF concentrations of S100B and classic AD biomarkers: Aβ42, t-tau in children with enteroviral meningitis (EM).

## Methods

### Study design

The study was conducted on children with meningitis caused by enteroviruses, who were hospitalized in the tertiary teaching hospital in Bialystok, Poland, during a single seasonal outbreak. The control group included children suspected of having a CNS infection, but evaluation of CSF ruled out the diagnosis.

### CSF measurements

CSF samples were centrifuged and stored at − 80 °C for analysis. Biomarker concentrations were measured with ELISA kits. Aβ42 was analyzed using INNOTEST β-AMYLOID(1-42) ELISA kit (Fujirebio Diagnostics, Malvern, PA, USA). Total tau levels were measured with INNOTEST h-tau ELISA kit (Fujirebio Diagnostics, Malvern, PA, USA). Protein S100B was analyzed with Human S100B ELISA kit (Elabscience, Bethesda, MD, USA). Values below the detection limit were estimated as the detection limit divided by two.

### Statistical methods

Statistical analysis was conducted using Statistica version 12 (TIBCO Software Inc.). Data are presented as median and interquartile range (IQR). Differences between the groups were analyzed using the Mann–Whitney *U* test. Correlations were calculated using Spearman’s rank correlation coefficient and adjusted using a general multiple regression model. A *p* value of less than 0.05 was considered statistically significant.

## Results

### Baseline characteristics

Forty-two children with EM were included in this study. The diagnosis of EM was confirmed by the presence of increased cell count in the CSF (> 5 cells/μL) and the detection of enterovirus RNA in CSF (*n* = 40) or stool samples (*n* = 2). Viral culture followed by sequencing revealed echovirus 30 to be a single cause of the outbreak. The baseline characteristics of the study groups and main results are presented in Table 1.

**Table 1:** Demographic, laboratory, and CSF characteristics of the studied groups

	Controls	EM
No. of patients	32	42
Age, years^*^	7.6 (5.1–13.5)	12.1 (7.0–15.2)
Female/male	13/19	12/30
CRP, mg/L^*^	4.8 (0.9–12.0)	5.4 (3.1–10.6)
Blood leukocyte count × 10^3^/μL^*^	9.7 (5.9–13.7)	9.8 (7.7–11.6)
CSF leukocyte count/μL^*^	1.0 (1.0–1.5)	109.0 (47.0–196.0)^**^
CSF protein, mg/dL^*^	10.0 (6.0–12.0)	27.5 (21.0–44.0)^**^
CSF mononuclear cells/μL^*^	–	63.6 (46.5–117.8)
CSF neutrophils/μL^*^	–	27.7 (5.9–106.1)
Aβ42, pg/mL^*^	1051.1 (737.6–1559.5)	459.4 (312.0–662.0)^**^
t-tau, pg/mL^*^	380.7 (233.5–518.6)	270 (164.8–396.2)
S100B, pg/mL^*^	350.2 (277.8–458.3)	329.9 (278.4–381.3)
t-tau/Aβ42 ratio^*^	0.32 (0.25–0.54)	0.54 (0.38–0.87)^**^

*EM*, enteroviral meningitis; *CRP*, C-reactive protein; *CSF*, cerebrospinal fluid; *Aβ42*, amyloid beta 42; *t-tau*, total tau; *S100B*, S100 calcium-binding protein B; ^*^Values expressed as median (interquartile range); ^**^p < 0.001

### Aβ_42_, t-tau, and S100B in enteroviral meningitis

**Fig. 1:**
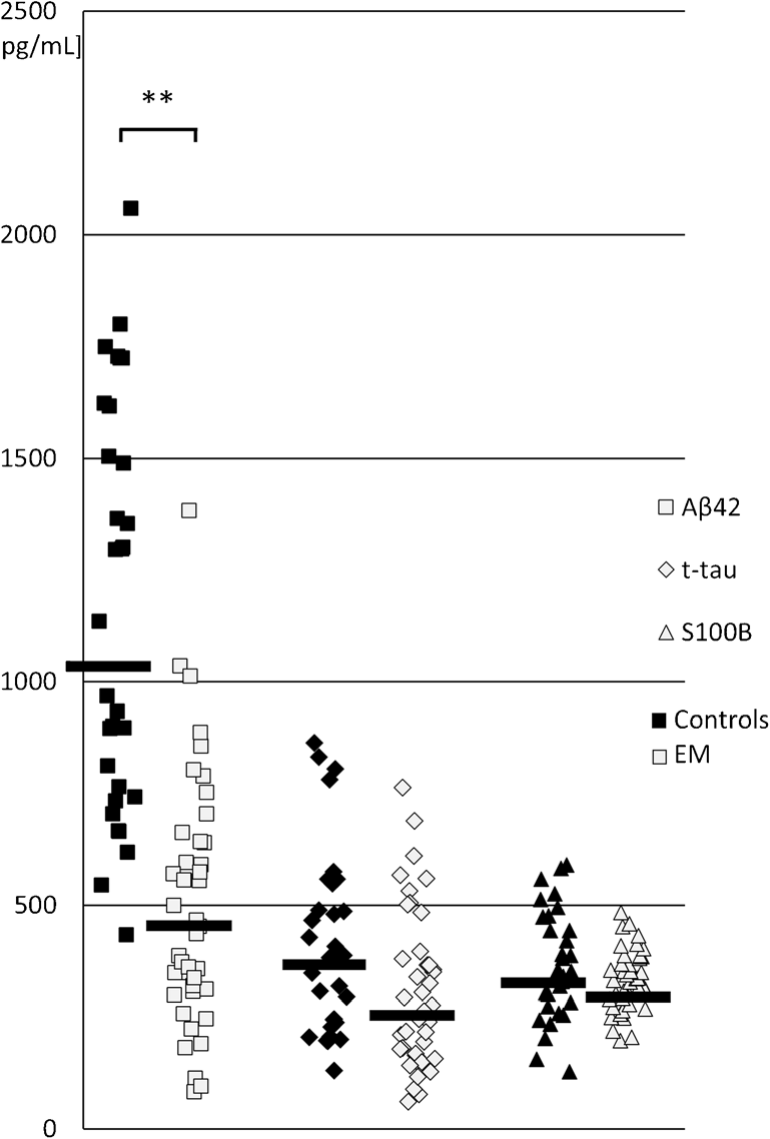
Scatter plot of cerebrospinal fluid concentrations of amyloid beta 42 (Aβ42, white square), total tau (t-tau, white diamond), and S100 calcium-binding protein B (S100B, white triangle) in enteroviral meningitis (EM) (white points) vs. controls (black points). Black horizontal lines represent median values. **p < 0.001

Concentration of Aβ42 in CSF was significantly reduced in EM patients. Interestingly, CSF concentrations of t-tau and S100B were not affected by the infection (Fig. 1). The ratio of CSF t-tau/Aβ_42_ increased in EM (Table 1).

### Correlations

**Fig. 2:**
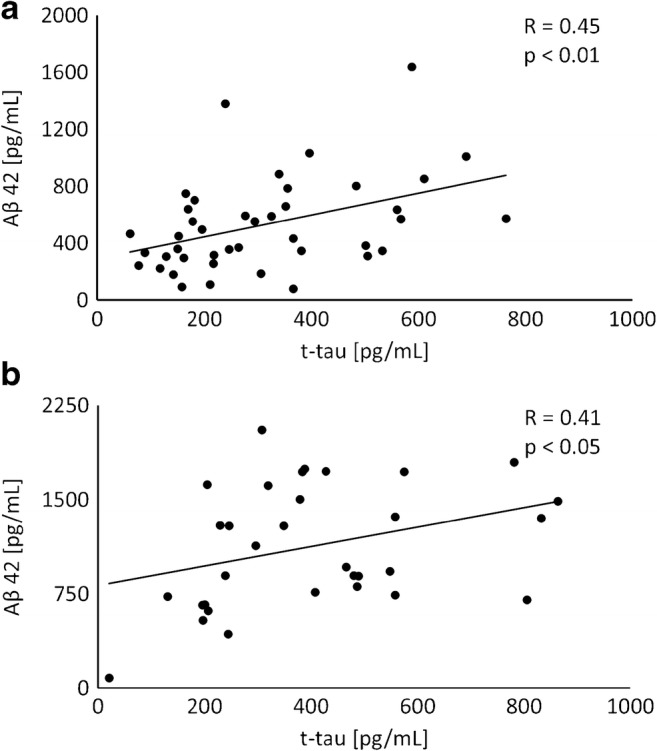
Relationships between cerebrospinal fluid (CSF) concentrations of amyloid beta 42 (Aβ42) and CSF total tau (t-tau) in enteroviral meningitis (**a**) and healthy controls (**b**). R, correlation coefficient

We did not find any significant correlations between the biomarkers and serum CRP, blood leukocytes, CSF leukocytes, or CSF protein concentration. However, CSF t-tau moderately correlated with Aβ42 in the EM group and controls (Fig. 2a, b). Correlations between S100B and two other biomarkers were insignificant. Age and gender were not related to the CSF biomarker profile.

**Fig. 3:**
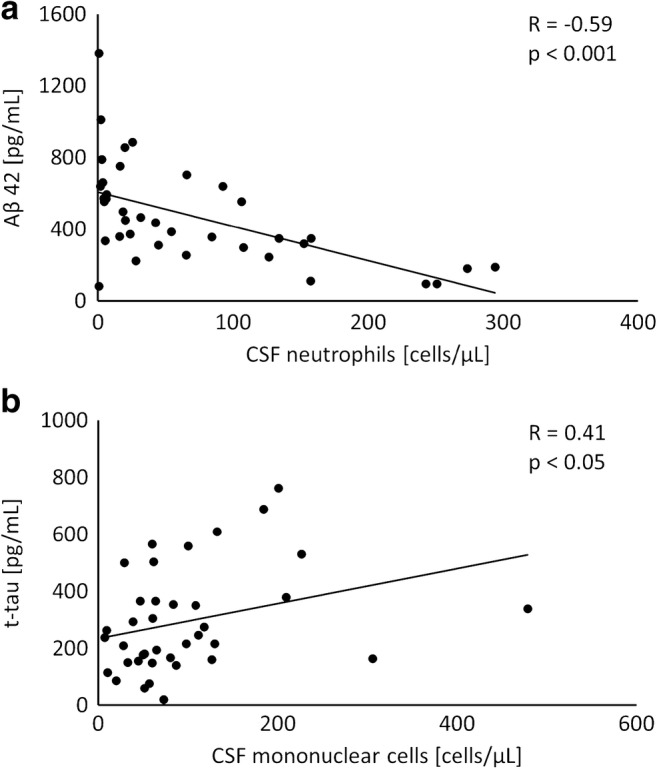
Relationships between (**a**) cerebrospinal fluid (CSF) absolute neutrophil count and CSF concentrations of amyloid beta 42 (Aβ42), and (**b**) between CSF absolute mononuclear cell count and total tau (t-tau) in enteroviral meningitis. R, correlation coefficient

The absolute number of neutrophils in CSF strongly and negatively correlated with CSF Aβ42 in EM (Fig. 3a). There was a positive correlation between CSF mononuclear cells and CSF t-tau (Fig. 3b). These correlations remained significant after adjustment for age, blood leukocytes, serum CRP, CSF cell count, and CSF protein concentration. Concentration of S100B did not correlate with neutrophils or with mononuclear cells in the CSF.

## Discussion

In this study, CSF Aβ42 concentration was decreased in EM. Similar observations were made in bacterial meningitis and HSV-1 encephalitis [9–11]. The decrease may be explained by a lower production of amyloid β peptides in the brain and disturbed clearance. The function of BBB is impaired in meningitis, allowing influx of proteins from the blood to the CSF. Possibly, serum proteins bind to Aβ42, affecting its detection; however, addition of serum to the CSF did not reduce Aβ42 levels in one study [10]. Additionally, Aβ42 has a direct antimicrobial activity and binds to various bacterial strains, what reduces its concentrations in CSF [12, 13].

Tau concentration in the CSF is a marker of brain parenchymal damage. Increased CSF tau levels are observed in brain hemorrhage, traumatic brain injury, severe bacterial meningitis, and HSV-1 encephalitis [9, 14, 15]. Meningitis, however, does not always result in increased CSF tau levels [10, 14, 16]. For example, HIV-associated dementia and neuroborreliosis were associated with a decrease in CSF t-tau [9, 17]. The extremely low levels of CSF t-tau were also observed in patients with Guillain-Barre Syndrome in one study. Authors suggest that the observed decrease may have been caused by increased elimination or consumption of tau protein, because its detection is not influenced by BBB dysfunction [14]. As it was shown by Süssmuth, CSF tau concentrations were increased in CNS infections with encephalitic complications only [14]. In our study, CSF t-tau levels were not increased in the EM. Protein S100B, a marker of glial cell injury, was not affected as well. Elevated S100B levels are observed in encephalitis and bacterial meningitis, but remain unaltered in viral meningitis [16, 18]. For example, CSF S100B levels over 960 pg/mL were associated with higher risk of brain injury in encephalitis of viral, bacterial, and unknown etiology [19]. Our results clearly indicate that meningitis caused by non-polio enteroviruses is not associated with a significant parenchymal damage.

We hypothesize that the correlations between CSF Aβ42, t-tau, neutrophils, and mononuclear cells may reflect the natural course of inflammatory response in EM. Neutrophils can predominate early in the course of aseptic meningitis [20]. As the number of neutrophils drops, the number of mononuclear cells increases. Mononuclear cells infiltrating CNS in EM may possibly cause a slight parenchymal damage in EM reflected in t-tau concentrations. It may also explain decreased Aβ42 levels, because blood-derived monocytes migrating into the CNS are key players in phagocytosis of Aβ [21].

Interestingly, the t-tau/Aβ42 ratio was higher in the EM group. The ratio over 0.52 was suggested to be a robust marker of an AD profile in the CSF [22]. Here, we show that an increased t-tau/Aβ42 ratio is not specific for AD and may also be observed in viral meningitis. We also found positive correlations between Aβ42 and t-tau concentrations in EM and healthy controls. This indicates that Aβ42 is linked to t-tau. A coupling of tau to Aβ oligomers in cognitively normal adults was reported before [23].

Several limitations of this study must be acknowledged. This is a cross-sectional study, so correlations we found do not necessary mean causation. This is, however, the first study to analyze CSF Aβ42, t-tau, and S100B concentrations in children with aseptic meningitis. The infection was caused by a single etiological agent making the study group homogenous, aiding in data interpretation.

## Conclusions

Our data suggest that there may be an important relation between cerebrospinal fluid leukocytes, Aβ42, and t-tau. Previous studies have already acknowledged that CNS infections have an impact on biomarkers of neurodegeneration in CSF, but failed to give insights on underlying processes. Here, we report that neutrophils may play a role in amyloid metabolism, and that mononuclear cells are linked to t-tau concentrations in children with enteroviral meningitis.
